# Supplementary material for: In Vitro Probiotic Properties and In Vivo Anti-Ageing Effects of Lactoplantibacillus plantarum PFA2018AU Strain Isolated from Carrots on Caenorhabditis elegans
Source: Microorganisms. 2023 Apr 21;11(4):1087. doi: 10.3390/microorganisms11041087 (PMC10144472; doi:10.3390/microorganisms11041087)
Supplement: Supplementary file 1 [file microorganisms-11-01087-s001.zip › microorganisms-2300545-supplementary.pdf]

Table S1. Primers for real time qPCR

Gene		Primer sequence
sek-1	FOR	5'-CAGAGCCGTTTATTGGGAAA
	REV	5'-TGCATCCGGCTTGTACAGT [1]
daf-2	FOR	5'-ATCGTCGGATTCTACTGTACTCCC
	REV	5'-CCGACATCTGACAATATTCATTCTCGT
sod-3	FOR	5′-AGAACCTTCAAAGGAGCTGATG
	REV	5′-CCGCAATAGTGATGTCAGAAAG
act-1	FOR	5′-GAGCGTGGTTACTCTTTCA
	REV	5′-CAGAGCTTCTCCTTGATGTC
gst-4	FOR	5'-TCAATGTGCCTTACGAGGATTA
	REV	5'-CGAATTGTTCTCCATCGACTTG
skn-1	FOR	5′-GTTCCCAACATCCAACTACG
	REV	5′-TGGAGTCTGACCAGTGGATT
daf-16	FOR	5′-TCAAGACCTCAAAGCCAATCAACTC
	REV	5′-ACGAGAAAGAAGGAGTAAGAGGAGG
